# Network Pharmacological Analysis and Experimental Validation of the Mechanisms of Action of Si-Ni-San Against Liver Fibrosis

**DOI:** 10.3389/fphar.2021.656115

**Published:** 2021-07-01

**Authors:** Siliang Wang, Cheng Tang, Heng Zhao, Peiliang Shen, Chao Lin, Yun Zhu, Dan Han

**Affiliations:** ^1^ Department of Pharmacy, Nanjing Drum Tower Hospital, The Affiliated Hospital of Nanjing University Medical School, Nanjing, China; ^2^ Department of Respiratory Medicine, Affiliated Hospital of Integrated Traditional Chinese and Western Medicine, Nanjing University of Chinese Medicine, Nanjing, China; ^3^ Department of Endocrinology, Jinling Hospital, Medical School of Nanjing University, Nanjing, China; ^4^ School of Pharmacy, School of Medicine and Holistic Integrative Medicine, Nanjing University of Chinese Medicine, Nanjing, China; ^5^ School of Medicine and Holistic Integrative Medicine, Nanjing University of Chinese Medicine, Nanjing, China

**Keywords:** network pharmacology, Si-Ni-San, liver fibrosis, inflammation, hepatic stellate cell, angiogenesis

## Abstract

**Background:** Si-Ni-San (SNS), a commonly used traditional Chinese medicine (TCM) formula, has potency against liver diseases, such as hepatitis and non-alcoholic fatty liver disease (NAFLD). However, the therapeutic efficacy and pharmacological mechanisms of action of SNS against liver fibrosis remain largely unclear.

**Methods:** A carbon tetrachloride (CCl_4_)-induced liver fibrosis mouse model was adopted for the first time to investigate the beneficial effects of SNS on liver fibrosis. The potential mechanisms of action of SNS were explored using the network pharmacology-based strategy and validated with the aid of diverse assays.

**Results:** SNS treatment reduced collagen and ECM deposition, downregulated fibrosis-related factor (hyaluronic acid and laminin) contents in serum, maintained the morphological structure of liver tissue, and improved liver function in the liver fibrosis model. Based on network pharmacology results, apoptosis, inflammation and angiogenesis, together with the associated pathways (including VEGF, TNF, caspase, PPAR-γ and NF-κB), were identified as the mechanisms underlying the effects of SNS on liver fibrosis. Further *in vivo* experiments validated the significant mitigatory effects of SNS on inflammatory infiltration and pro-inflammatory cytokine contents (IFNγ, IL-1β and TGF-β1) in liver tissues of mice with liver fibrosis. SNS suppressed pathologic neovascularization as well as levels of VEGFR1, VEGF and VEGFR2 in liver tissues. SNS treatment additionally inhibited hepatic parenchyma cell apoptosis in liver tissues of mice with liver fibrosis and regulated apoptin expression while protecting L02 cells against apoptosis induced by TNF-α and Act D *in vitro*. Activation of hepatic stellate cells was suppressed and the balance between MMP13 and TIMP1 maintained *in vitro* by SNS. These activities may be associated with SNS-induced NF-κB suppression and PPAR-γ activation.

**Conclusion:** SNS effectively impedes liver fibrosis progression through alleviating inflammation, ECM accumulation, aberrant angiogenesis and apoptosis of hepatic parenchymal cells along with inhibiting activation of hepatic stellate cells through effects on multiple targets and may thus serve as a novel therapeutic regimen for this condition.

## Introduction

Liver fibrosis refers to histological changes induced by chronic inflammation resulting from multiple acute and chronic liver conditions, including infection with viral hepatitis B or C virus (HBV or HCV), alcoholic steatohepatitis, non-alcoholic steatohepatitis (NASH), non-alcoholic fatty liver disease (NAFLD), cholestatic liver disease and biliary disease (Koyama and Brenner, 2017; Mendez-Sanchez et al., 2020; Sterling et al., 2020). Liver injury can cause hepatic stellate cell (HSC) hyperactivity and promote extracellular matrix (ECM) accumulation (Cai et al., 2020). In this case, collagen fibers accumulate within the hepatocyte extracellular space, causing loss of blood supply to hepatocytes (Kisseleva and Brenner, 2020; Meurer et al., 2020). In the absence of appropriate treatment, liver fibrosis can progress to cirrhosis or even liver failure (He et al., 2015). Due to the reversibility of this condition, interventions can effectively improve clinical outcome, even at an advanced stage of liver fibrosis (Koyama et al., 2016). Recent treatments have mainly aimed to inactivate HSCs, protect against apoptosis of hepatic parenchymal cells (HPC), control inflammation and regulate metabolism of the extracellular matrix (ECM) (Hou et al., 2015; Zhou et al., 2019; Chan et al., 2020; Lambrecht et al., 2020).

Few potent antifibrotic drugs have been extensively validated clinically or applied as therapy to date. The existing antifibrotic drugs are limited in that insufficient quantities are absorbed *via* activated HSCs and significant side-effects are generated including hepatotoxicity or tumor occurrence (Fagone et al., 2015; Ma et al., 2017). Therefore, more research attention should be paid to natural herbs, such as traditional Chinese medicine (TCM) formulae, that may provide greater opportunities to prevent and treat chronic liver diseases without inducing side-effects (Song et al., 2019; Wang T. et al., 2020). TCM formulae generally incorporate complex combinations of natural herbs, animal products and minerals, consequently serving as multifunctional therapeutic agents that exert their effects in a holistic manner (Cheung et al., 2012; Gong, 2012; Liu, 2016). Numerous TCM-based formulae and herbal extracts, such as silymarin, emodin and curcumin, have been shown to protect against liver fibrosis through inhibiting inflammation, promoting ECM decomposition, and suppressing activation of HSCs (Zhao et al., 2017; Cao et al., 2018; Liu et al., 2018; Zhao et al., 2018; Jiang et al., 2020).

Si-Ni-San (SNS), a noted TCM formula used for thousands of years in China for coordinating liver and spleen functions, was initially documented by Zhong-Jing Zhang in the Eastern Han Dynasty (Jiang et al., 2003). SNS comprises four herbal medicines: Bupleuri Radix (*Bupleurum chinense* DC., *Bupleurum scorzonerifolium* Willd., Chaihu, CH), Paeoniae Radix Alba *(Paeonia lactiflora* Pall., Baishao, BS), Aurantii Fructus Immaturus (*Citrus × aurantium* L., Zhishi, ZS), and Glycyrrhizae Radix et Rhizoma (*Glycyrrhiza glabra* L., *Glycyrrhiza inflata* Batalin, *Glycyrrhiza uralensis* Fisch. ex DC., Gancao, GC) at a ratio of 1:1:1:1. SNS is reported to be efficient in improving “stagnation of Qi due to depression of the liver” in TCM theory and adopted clinically to alleviate several liver diseases, including NAFLD and hepatitis (Zhu et al., 2019). Accumulating studies suggest that the active herbal components of SNS, such as Paeoniflorin in BS, Saikosaponin A in CH, hesperidin in ZS and Liquiritigenin in GC, exert diverse pharmacological effects, such as anti-inflammation, anti-fibrosis, anti-HSC activation and liver protection activities (Zhao et al., 2014; El-Sisi et al., 2017; Geng et al., 2020; Shiu et al., 2020). However, the specific roles and protective mechanisms of action of SNS against liver fibrosis remain unclear.

The TCM formula is characterized by multiple components and targets. Traditional experimental approaches have been unsuccessful in characterizing the underlying complex pharmacological mechanisms. Moreover, due to the current lack of knowledge on specific activities and mechanisms, TCM is not commonly employed on a global scale (Guo et al., 2020). To ensure effective clinical application, the scientific foundation and mechanisms underlying the beneficial effects of TCM require elucidation. Owing to rapid developments in bioinformatics and systems biology approaches, network pharmacology-based analysis presents novel tools that could aid in clarifying the complex pathways of TCM, such as SymMap (Wu et al., 2019) and TCMSP (Zhang R. et al., 2019). Holism is a critical principle of TCM. Integration of network science with abundant TCM experience may therefore help to transform the research pattern from a single drug target to multi-component network targets, which should provide novel insights into the activities and associated mechanisms of the herbal components of TCM (Pun and Chor, 2020). In the present study, we examined the active components and mechanisms underlying the effects of SNS on liver fibrosis using network pharmacology analysis in combination with experimental validation, which were carried out in accordance with Network Pharmacology Evaluation Method Guidance-Draft (Li, 2021).

## Methods

### Preparation of Si-Ni-San Experimental Agents

Crude TCM herbs (dried roots of *Bupleurum scorzonerifolium* Willd., dried roots of *Paeonia lactiflora* Pall., dried immature fruits of *Citrus × aurantium* L., dried roots and rhizomes of *Glycyrrhiza uralensis* Fisch. ex DC.) were provided by Beijing Tongrentang. In brief, CH, BS, ZS, and GC (25 g each) were extracted twice for 60 min each using boiling water (1:6 and 1:4, w/v), followed by filtering and mixing. Afterward, we concentrated the filtrates by reducing pressure and then on an electric thermostatic water bath at 70°C to thick paste [0.8 kg (crude medicine)/L]. Further, the SNS experimental powders were producted through freeze-drying technology, and the process was shown below: refrigeration at −40°C, cryopreservation for 2 h at −20°C, cryopreservation for 16 h at −10°C, drying at 20°C for 5 h, and secondary drying at 35°C for 2 h. The contents of the four major components of the freeze-dried powder were quantified *via* HPLC (Table 1 and the HPLC chromatograms were shown in Supplementary Figure S1).

**TABLE 1:** The contents of four major ingredients in the freeze-dried powder.

Ingredients	Contents in mg/g
Glycyrrhizic acid	128.3 ± 0.04
Saikosaponin A	91.5 ± 0.02
Paeoniflorin	389.7 ± 0.03
Naringin	134.7 ± 0.05

### Animals and Treatment

Male C57BL/6J mice (7 weeks old, 18–20 g) obtained from Beijing Vital River Laboratory Animal Technology Co. Ltd. (Beijing, China) were subjected to a temperature-, light-, and humidity-controlled environment. All animals had free access to food and water and were allowed to acclimatize to the conditions for one week prior to experiments. Experimental protocols were approved by the Animal Care and Use Committee of Nanjing University of Chinese Medicine (Nanjing, China) and conducted according to the Guidelines for the Care and Use of Laboratory Animals (ACU200905, 29th September 2020). Liver fibrosis was induced through intraperitoneal injection with 4% (v/v) carbon tetrachloride (CCl_4_) in olive oil at a dose of 5 ml/kg body weight (BW) twice weekly for eight consecutive weeks (Cao et al., 2018). An appropriate amount of SNS freeze-dried powders was collected, prepared into the 500 g (powder)/L solution with distilled water, and used for intragastric administration of experimental animals. The 32 mice were classified into four groups for 8 weeks of treatments as follows: 1) Control group given gavage of distilled water, 2) Model group with liver fibrosis given gavage of distilled water, 3) SNS low-dose (SNS-L) group with liver fibrosis given gavage of SNS at 5 g (powder)/kg BW, and 4) SNS high-dose (SNS-H) group with liver fibrosis given gavage of SNS at 10 g (powder)/kg BW. The BW of individual mice from each group was recorded once weekly for eight consecutive weeks.

### Histomorphology Assay

Liver tissues were processed with 10% formalin fixation followed by paraffin embedding, slicing into 5 µm sections, and hematoxylin-eosin (HE) staining for histopathological analysis or Masson trichrome dye and Sirius red staining to examine collagen formation. An upright microscope was utilized to observe histological sections and obtain photographic images. Subsequently, 10 fields of view (FOV) were selected from each sample from all treatment groups for observation. Areas with positive Sirius red or Masson staining were evaluated using Aperio ImageScope-Pathology Slide Viewing Software. Liver fibrosis was graded using a previously reported fibrosis staging system (Goodman, 2007) (Supplementary Table S1).

### Network Pharmacology Analysis

*Data preparation:* Data on each herbal component of SNS were collected using the Traditional Chinese Medicine System Pharmacology Database (TCMSP, http://lsp.nwu.edu.cn/tcmsp) (Ru et al., 2014), Traditional Chinese Medicines Integrated Database (TCMID, http://www.megabionet.org/tcmid/) (Huang et al., 2018), and associated literature. The data acquired were uploaded to the ingredient database.

*Oral bioavailability (OB) and drug-likeness screening:* OB prescreening indicates the distribution level of an oral drug dose in bloodstream, which is a critical prerequisite for identification and application of oral drugs in the clinic. Drug-likeness is a qualitative concept used for evaluating the structural similarities of compounds with therapeutic efficacy in the Drugbank database, which can be determined immediately following drug discovery. Wang and colleagues reported the calculations for the two above parameters in detail (Xu et al., 2012; Tao et al., 2013). At last, in line with the TCMSP database recommendations, an OB of 30%, together with a drug-likeness index of 0.18, (mean value for all molecules within the DrugBank database) (Wang X. et al., 2012), was adopted as the cut-off value for selecting the potential pharmacodynamic components.

*Prediction of putative drug targets for SNS:* Identification of targets is suggested to be the critical link during drug discovery. In our study, the systemic drug targeting method of Wang and co-workers was adopted for precise determination of potential targets for the therapeutic components of SNS (Li et al., 2014). As for the present systemic drug targeting method, it is conducted from two levels, and it represents a systemic target predicting approach to integrate several algorithms. 1) The HIT database was applied in collection and retrieval of the drug-target interactions verified in experiments. 2) The SysDT model was used to predict the putative compound targets that have not been validated in experiments, and high sensitivity, specificity, and consistency were attained in the prediction of drug-target interactions.

*Collection of known liver fibrosis-related targets:* Targets known to be related to liver fibrosis were mainly collected based on five resources: 1) MalaCards human disease database (https://www.malacards.org) (Rappaport et al., 2017), 2) OMIM database (http://www.omim.org) (Amberger et al., 2019), 3) Therapeutic Target Database (TTD, http://bidd.nus.edu.sg/group/cjttd/) (Zhu et al., 2010), 4) DrugBank database (https://www.drugbank.ca) (Wishart et al., 2008), and 5) the Genetic Association Database (GAD, https://geneticassociationdb.nih.gov) (Becker et al., 2004). After the redundant targets were deleted, known targets related to liver fibrosis were screened.

*GO-BP and KEGG enrichment analyses:* Targets were annotated based on the Omicshare (http://www.omicshare.com/tools) database for further clarification of the corresponding functions. Differences of ≤0.05 indicated statistical significance and the enriched terms of GO and KEGG analyses were subsequently determined *via* hypergeometric examination.

### Serum Biochemical and Cytokine Analysis

At 24 h following the final injection, 0.8 ml peripheral blood was obtained from every mouse through eyeball enucleation. After 60 min of incubation at ambient temperature, blood samples were centrifuged for 10 min at 3,000 rpm and 4°C to separate serum. After the addition of protein extraction solution, samples were incubated for 30 min on ice and centrifuged at 15,000 rpm to collect supernatant fractions that were stored at −20°C for quantification of protein contents. ELISA kits were utilized to determine the serum levels of laminin (LN; GeneTex, cat no. GTX37121), hyaluronic acid (HA; Abbexa, cat no. abx156663), albumin (Abcam, cat no. ab179887), AST (Nanjing Jiancheng Bio, cat no. C010-2-1), ALT (Nanjing Jiancheng Bio, cat no. C009-2-1), tumor necrosis factor (TNF)-α (R&D systems, cat no. MTA00B), TGF-β1 (R&D systems, cat no. MB100B), interleukin (IL)-1β (R&D systems, cat no. MLB00C) and interferon (IFN)-γ (R&D systems, cat no. MIF00) according to the manufacturers’ protocols. The BioTek Synergy instrument was applied for reading optical density (OD) at specific wavelengths.

### Examination of Alanine Aminotransferase, Aspartate Aminotransferase, Tumor Necrosis Factor-Alpha, Hydroxyproline and Vascular Endothelial Growth Factor in Liver

Briefly, 150 µL ice-cold lysis buffer was added to homogenize 15 mg liver tissue. The mixture was centrifuged for 15 min at 10,000 rpm and 4°C to collect the supernatant fractions, which were stored at −20°C for quantification of protein levels. ELISA kits were utilized to determine the tissue contents of ALT, AST, TNF-α, hydroxyproline (Hyp, Nanjing Jiancheng Bio, cat no. A030-2-1) and VEGF-A (Invitrogen, cat no. BMS619-2) according to the manufacturers’ protocols.

### Immunofluorescence Analysis

Immunofluorescence double staining was performed for determination of CD34 expression. Tissue sections were stained with primary anti-CD34 antibody (Abcam, cat no. ab81289), followed by the appropriate secondary goat anti-rabbit IgG (H + L)-FITC antibody (1:200; Bioworld, cat no. BS10950). Finally, sections were mounted with mounting medium for fluorescence with 4′, 6′-diamidino-2-phenylindole (DAPI; Beyotime, cat no. C1002). Fluorescence images were analyzed using ZEN pro 2012 imaging software on a Zeiss inverted microscope.

### Terminal Deoxynucleotidyl Transferase-Mediated dUPT Nick-End Labeling Assay

After deparaffinization and rehydration, sections were subjected to the TUNEL assay to detect apoptotic hepatocytes using an Apoptosis Detection Kit (Beyotime, cat no. C1098). Each section was incubated for 15 min with proteinase K at 37°C and 3% hydrogen peroxide added to block endogenous peroxidase activity. After reaction with deoxynucleotidyl transferase, sections were incubated with the anti-digoxigenin conjugate prior to DAB color development. Next, each section was subjected to 0.5% Methyl Green counterstaining and mounting. Five high-power fields of view (FOV) were selected for each section and examined using Image Pro Plus software (Media Cybernetics, Inc., Rockville, MD, United States). The percentage of positively stained cells (apoptotic index) was determined according to the formula: apoptotic index = positive cell number/total cell number × 100%.

### Cell Culture

LX2 and L02 cells purchased from the Type Culture Collection of the Chinese Academy of Sciences (Shanghai, China) were used for experimental validation *in vitro*. The human HSC cell line, LX2, was cultivated in RPMI 1640 (Gibco) containing 10% fetal bovine serum (FBS, Gibco). Normal human hepatic L02 cells were cultivated in DMEM (Gibco) containing 10% FBS. The cell culture media contained 100 mg/L streptomycin and 100,000 U/L penicillin. Both cell lines were cultivated under 37°C and 95% O_2_/5% CO_2_.

For examination of the roles and mechanisms of action of SNS in activated LX2 cells, 5 ng/ml TGF-β1 was used to stimulate quiescent LX2 cells for 24 h in the presence or absence of SNS (50 mg/L). Cells were subsequently harvested to detect related items.

### Cell Viability Assay

After digestion, counting and seeding of L02 cells into 96-well plates at a density of 3 × 10^3^ cells/plate for 12 h, fresh medium containing various concentrations of SNS or distilled water was used to replace the original medium for 18 h, followed by incubation of cells with 200 ng/ml Act D and 20 ng/ml TNF-α for 6 h. The MTT assay was conducted at a wavelength of 490 nm to evaluate cell proliferation.

### Cell Apoptosis Assay

In the apoptosis assay, cells (3.0 × 10^5^ cells/well) were inoculated in 6-well plates. After treatment with 50 mg/L and 100 mg/L SNS for 42 h, L02 cells were incubated for 6 h with 20 ng/ml TNF-α (Peprotech, cat no. 300-01A) and 200 ng/ml Act D (Merck, cat no. 50-76-0). The cell apoptosis rate was analyzed with the Muse Annexin V and Dead Cell Assay (Millipore, cat no. MCH100105) in keeping with the manufacturer's instructions. All assays were conducted in triplicate.

### RNA Isolation and qRT-PCR Analysis

RT-PCR analysis was performed to determine collagen I, α-SMA, fibronectin, VEGFR1, VEGFR2, VEGF, TNF-α, TGF-β1, IL-1β, IFN-γ, TIMP and MMP13 mRNA levels in liver tissue or LX2 cells. Specifically, TRIzol reagent (Invitrogen, cat no. 15596018) was utilized to isolate total RNA from LX2 cells or liver tissues under RNase-free conditions. The resultant total RNA (1 mg) was used to prepare cDNA *via* reverse transcription using Hiscript^®^ II QRTSuperMix (Vazyme, cat no. R223-01) and gDNA Eraser, as recommended by the manufacturer. Quantitative RT-PCR with the SYBR Green Master kit (Bio-Rad) was utilized to quantify specific transcripts and the ABI 7500 RT-PCR system (Applied Biosystems) employed for detection and analysis. All gene-specific primers were prepared by Sangon Biotech. The primer sequences used are presented in Supplementary Table S2.

The mRNA expression levels were normalized to β-actin control. The PCR conditions were as follows: 30 s under 95°C, 40 cycles of 5 s under 95°C, and 30 s under 60°C. The comparative CT approach was used to calculate relative levels of mRNA.

### Western Blot

Western blot analysis was performed to determine protein expression of collagen I, α-SMA,VEGF, fibrolectin, KDR, TNF-α, TGF-β1, IFN-γ, IL-1β, TIMP, MMP13, cleaved caspase 3, Bax, Bcl2, and GAPDH in LX2 or L02 cells or liver tissue. Specifically, lysis buffer (Beyotime, cat no. P0013C) containing 1 nM phenylmethanesulfonyl fluoride (PMSF) (Beyotime, cat no. ST505) was used to prepare tissue homogenates. After denaturation, proteins were subjected to 8–12% bis-Tris/polyacrylamide gel electrophoresis (Beyotime, cat no. P0690) and subsequently transferred to polyvinylidene fluoride (PVDF) membranes (Amresco). Membranes were coated with Millipore filter (pore size 0.45 mm) and blocked for 2 h using TBST blocking solution containing 0.1% Tween-20 and 5% skimmed milk powder. Next, membranes was subjected to overnight incubation with the appropriate antibodies (Abcam; anti-collagen I, cat no. ab260043; anti-alpha-SMA, cat no. ab124964; anti-fibronectin, cat no. ab268020; anti-VEGFR2, cat no. ab115805; anti-VEGFR1, cat no. ab32152; anti-TIMP1, cat no. ab211926; anti-MMP13, cat no. ab219620; anti-cleaved caspase 3, cat no. ab32042; anti-Bax, cat no. ab32503; anti-Bcl-2, cat no. ab182858; anti-caspase 8, cat no. ab3239; anti-PPARγ, cat no. ab178866; anti-p65, cat no. ab32536 and anti-GAPDH, cat no. ab181602) diluted in TBST (1:1,000) containing 5% bovine serum albumin (BSA) at 4°C. Each membrane was further incubated for 2 h with horseradish peroxidase (HRP)-conjugated secondary antibody (Abcam, cat no. ab6721) diluted in TBST (1:10,000) containing 5% skimmed milk powder at ambient temperature. An enhanced chemiluminescence kit (Applygen Technologies) was employed to detect immunoreactivity. Image-Pro Plus software (version 6.0) was adopted for blot scanning and analysis of band intensity. Band intensity was determined according to the formula: band intensity = sum of all pixel values in a selected segment—background pixel value in the segment.

### Statistical Analysis

Data are presented as means ± SD. Student’s test was utilized to assess the differences in related parameters between experimental and control groups. One-way ANOVA was adopted to assess differences among several groups and Tukey’s test used to determine significance. *p* values <0.05 indicated significant differences. GraphPad Prism software (version 6.0) was employed for statistical analysis.

## Results

### Si-Ni-San Attenuates CCl_4_-Induced Hepatic Pathological Changes and Dysfunction

**FIGURE 1:**
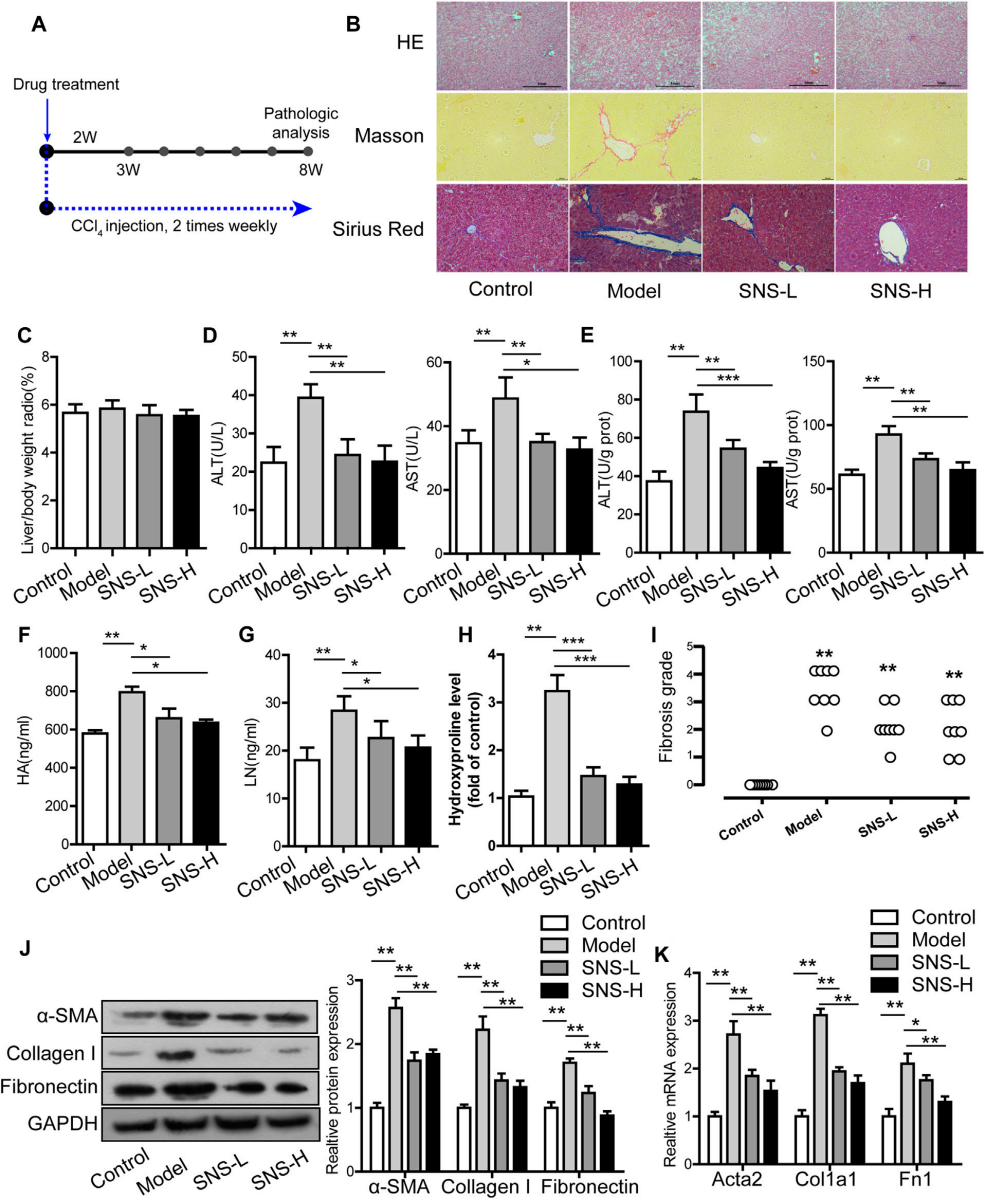
SNS ameliorates hepatic fibrosis in CCl_4_-treated mice. **(A)** Flowchart of animal experiments. **(B)** Representative images of liver sections stained with H&E or Masson or Sirius red (magnification, 100×). **(C)** Ratio of liver weight to body weight. **(D)** Concentrations of ALT and AST in serum. **(E)** Concentrations of ALT and AST in liver homogenates. **(F)** Concentrations of HA in serum. **(G)** Concentrations of LN in serum. **(H)** Effect of SNS on liver hydroxyproline. **(I)** Effect of SNS on fibrosis grading score. **(J)** Effects of SNS on protein levels of α-SMA, collagen I and fibronectin in liver tissue. **(K)** Effects of SNS on mRNA levels of Acta2, Col1a1, and Fn1 in liver tissue. Data are presented as mean ± SD (*n* = 6). *p < 0.05, **p < 0.01, ***p < 0.001 compared to control or model group.

The CCl_4_-induced liver fibrosis mouse model was used due to its convenient time frame (Figure 1A). H&E-stained liver tissue sections were subjected to microscopy analysis. As shown in Figure 1B, intact hepatic lobules were evident in liver tissues of the blank group along with normal hepatic sinusoids and orderly arranged hepatic cell cords and no hyperplasia of collagen fibers or infiltration of inflammatory cells. In the model group, liver tissues displayed obscure hepatic lobule structure along with destroyed hepatic cell cords, slight cell swelling, necrosis and fatty degeneration, infiltration of inflammatory cells and fibrocytes, which were partially mitigated after SNS treatment. The regulatory effects of SNS on alanine aminotransferase (ALT) and aspartate aminotransferase (AST) in liver tissues and sera of mice with liver fibrosis were further examined. SNS treatment induced a remarkable decrease in the ALT and AST contents relative to the model group (*p* < 0.01) but had no obvious influence on the ratio of liver weight to body weight (Figures 1C–E).

### Si-Ni-San Alleviates Liver Fibrosis Induced by CCl_4_

We initially examined the serum levels of factors related to liver fibrosis, such as hyaluronic acid (HA) and laminin (LN). As illustrated in Figure 1F,G, HA and LN levels were slightly increased in the model group compared to the control group, but markedly declined in the SNS group relative to the model group (*p* < 0.01).

The regulatory effects of SNS on collagen and ECM deposition in the liver were examined by visualization through Sirius red and Masson staining and measured based on Hyp and Col-I levels in liver and fibrosis grading scores. Under conditions of CCl_4_ induction, collagen was deposited in Sirius red and Masson-stained sections, particularly the perisinusoidal areas and diverse pseudo lobules. In the presence of SNS, the number of pseudo lobules and collagen deposition was decreased (Figure 1B). The Hyp levels in liver tissue and fibrosis grading scores were markedly elevated in CCl_4_-stimulated mice relative to the control group and conversely reduced in the SNS-treated group (Figures 1H,I). CCl_4_ treatment triggered an increase in Col-I mRNA and protein expression while SNS exposure had the opposite effect (Figures 1J,K).

We further explored the potential role of SNS in HSC activation. Liver tissues stimulated with CCl_4_ showed significantly increased mRNA and protein expression of α-SMA and fibronectin, indicative of activated HSCs. SNS exposure led to significant attenuation of α-SMA and fibronectin expression induced by CCl_4_, clearly signifying suppression of HSC activation in fibrotic liver (Figures 1J,K).

### Network Pharmacological Analysis of Si-Ni-San for Liver Fibrosis

#### Screening of Potential Pharmacodynamic Components and Targets of Si-Ni-San

**FIGURE 2:**
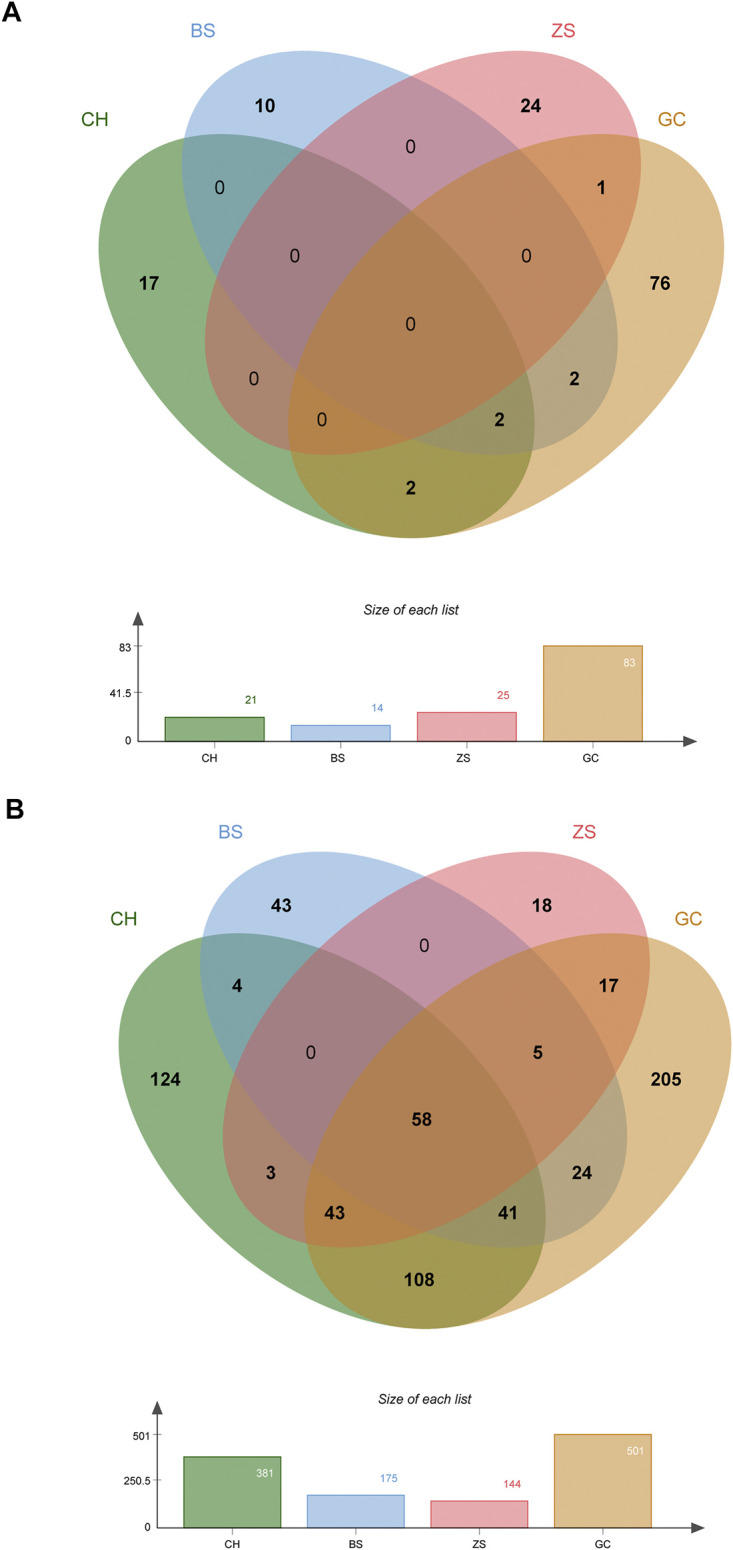
Screening of potential pharmacodynamic components and targets of SNS. **(A)** Venn diagram of potential pharmacodynamic components in each herb of SNS. **(B)** Venn diagram of potential pharmacodynamic targets of each herb of SNS.

Despite several studies on the therapeutic mechanisms of action of TCM, limited progress has been reported to date. Currently, no specific and efficient techniques for identification of the active components of herbal medicines are available. One promising approach involves integration of OB screening with drug-likeness evaluation. In the present study, 134 compounds with suitable OB and drug-likeness values were identified as potential pharmacodynamic ingredients of SNS (Supplementary Table S3). Specifically, 21, 14, 25, and 83 candidate compounds were detected in CH, BS, ZS, and GC, respectively (Figure 2A). Among these, kaempferol, oleanolic acid, isorhamnetin, quercetin, mairin, sitosterol and naringenin have been identified in the different herbal components of SNS and their biological activities extensively characterized. For example, kaempferol in CH, BS, and GC has widespread pharmacological activities, such as anti-fibrosis and anti-inflammation effects (Xu et al., 2019; Bian et al., 2020). Naringenin, widely detected in ZS and GC, exerts diverse pharmacological effects and is involved in modulation of several signaling pathways, such as pathologic angiogenesis-related VEGF/KDR and inflammatory reaction-related NLRP3/NF-κB (Li Q. et al., 2016; Fan et al., 2017).

Overall, TCM prescriptions exert widespread therapeutic effects on complex diseases that are totally dependent on the synergistic actions of multiple compounds on diverse targets. Accordingly, in addition to uncovering the pharmacodynamic components of SNS, it is necessary to explore the potential therapeutic targets. In this study, genomic, chemical and pharmacological data were integrated with a view to predicting the potential targets of the pharmacodynamic components of SNS. In total, 693 potential targets were screened for 134 pharmacodynamic ingredients (Supplementary Table S4), among which, 381, 175, 144, and 501 were associated with CH, BS, ZS and GC, respectively (Figure 2B). While all four herb components had diverse targets, several common targets were additionally identified. Moreover, similar activities were observed for different herb components of SNS, which were possibly attributable to their regulation of shared targets. For example, both CH and BS have been shown to reverse acute and chronic liver injury induced by various irritants (Wang R. et al., 2012; Wang Y.-X. et al., 2019).

#### Mining of Core Targets and Core Components of Si-Ni-San Associated With Liver Fibrosis

**FIGURE 3:**
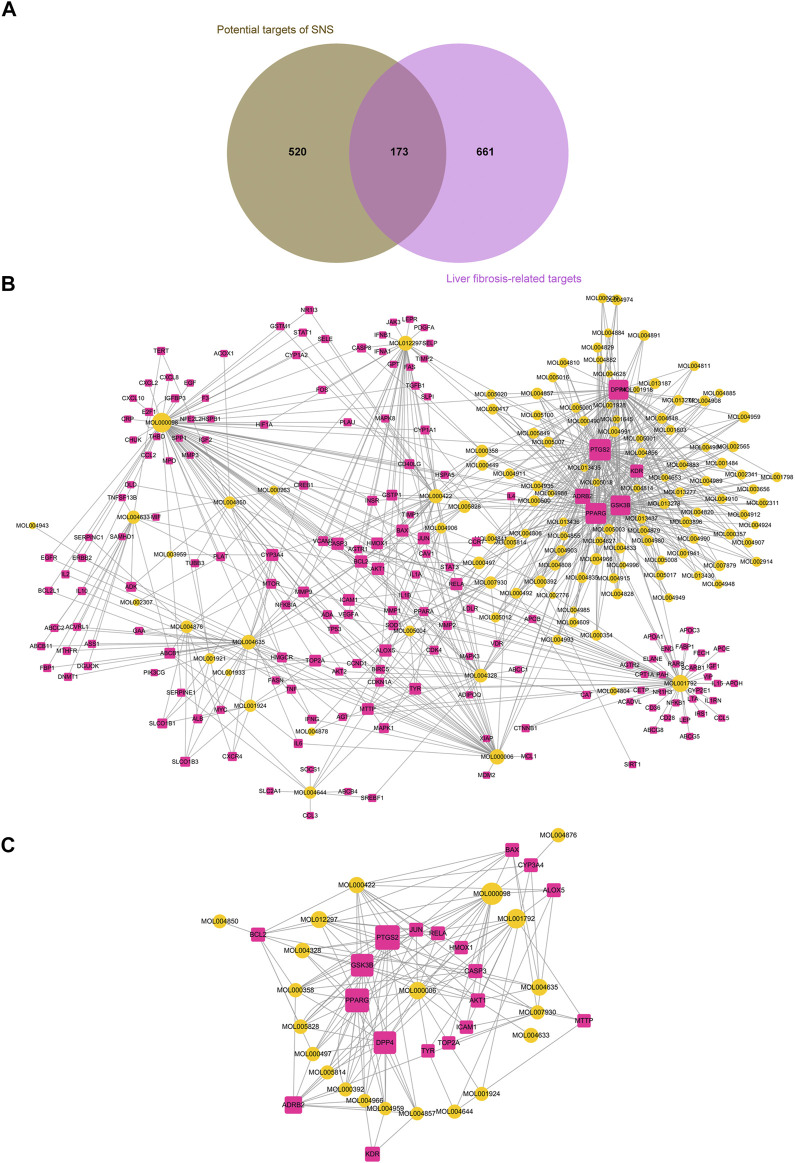
Excavation of the core pharmacodynamic components and targets of SNS with activity against liver fibrosis. **(A)** Venn diagram showing that SNS shares 173 potential targets with known pathological targets of liver fibrosis. **(B)** Candidate active ingredient-target network of SNS. **(C)** Core active ingredient-target network of SNS.

Liver fibrosis is a polygenic predisposing disease. Determination of the interactions between genes and the environment may aid in illustrating the pathogenesis of liver fibrosis. In our experiments, 834 targets related to liver fibrosis were identified from five resources (Supplementary Table S5). Among these, some were also potential targets of pharmacodynamic components of SNS. Overall, 173 candidate targets of SNS for activity against liver fibrosis were predicted (Supplementary Table S6, Figure 3A) and the related pharmacodynamic ingredients (*n* = 119) considered the candidate pharmacological components underlying the therapeutic effects of SNS.

Cytoscape was adopted for constructing the candidate component-target network of SNS (Figure 3B). To improve screening of the core components and targets of SNS responsible for therapeutic activity against liver fibrosis, we utilized the Cytohubba plug-in (Chin et al., 2014) for calculating and sorting the node topological parameters (degrees) in the constructed candidate component-target network (Supplementary Table S7). Nodes with a degree greater than or equal to two-fold median degree values (=6) for all nodes in the network were selected as the core active ingredients (*n* = 21, Supplementary Table S8) and core targets (*n* = 19, Supplementary Table S9) of SNS and used to establish the core component-target network (Figure 3C). Interestingly, all the core active ingredients with the top 5 degree values (quercetin, liquiritigenin, luteolin, puerarin, saikosaponin A) have been shown to reverse the occurrence and development of liver fibrosis through diverse pathways (Li et al., 2015; Chen et al., 2017; Hou et al., 2018; Lee et al., 2019; Zhang Q. et al., 2019).

#### Enrichment Analysis of Core Targets of Si-Ni-San

**FIGURE 4:**
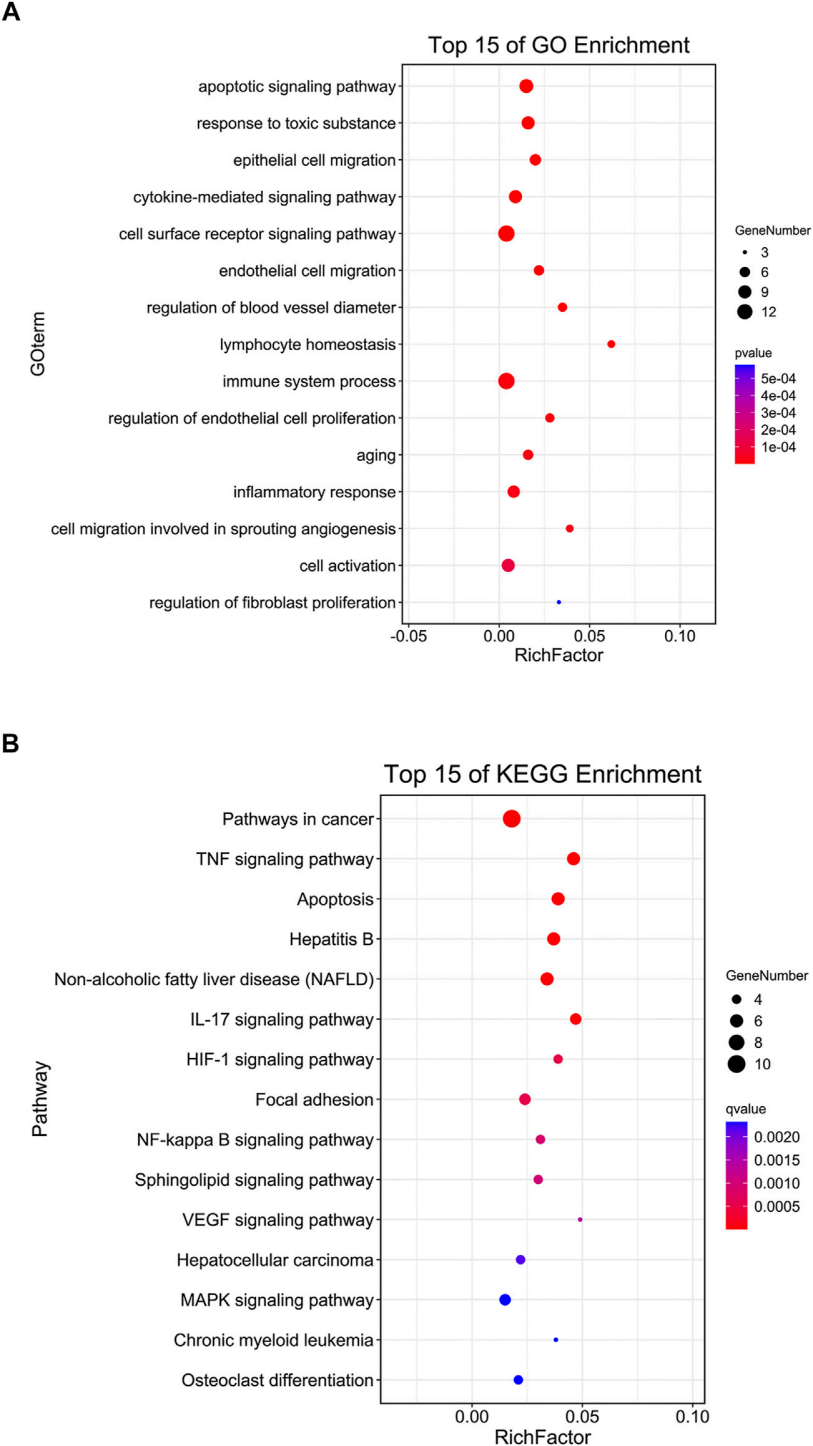
Enrichment analysis of core targets of SNS in treating liver fibrosis based on Omicshare. A *p*-value cut-off of ≤0.05 was considered significant and the hyper-geometric test applied to identify enriched GO-BP terms **(A)** and KEGG pathways **(B)**.

For clarification of the multi-target and multi-pathway mechanisms underlying the effects of SNS on liver fibrosis, the Omicshare online tool was utilized to conduct GO biological process (BP) and KEGG pathway analyses for the 19 core targets screened based on their degree values, with the aim of identifying biological processes (BPs) and signal transduction pathways of SNS associated with liver fibrosis (*p* < 0.05, FDR <0.05). Apoptosis, angiogenesis-related proliferation and migration of endothelial cells and immuno-inflammatory responses were the GO-BP items displaying the most significant enrichment (Figure 4A). Moreover, the core targets were predominantly related to several KEGG pathways, such as apoptosis-related, liver disease (NAFLD, hepatitis B and hepatocellular carcinoma)-related, TNF, VEGF, NF-κB, and MAPK pathways (Figure 4B), implicating their involvement in the effects of SNS on liver fibrosis. Subsequently, tissue and serum samples from the *in vivo* CCl_4_-induced liver fibrosis mouse model and a series of experiments *in vitro* were employed to validate the biological processes and signal transduction pathways identified based on the results of network pharmacological analysis.

### Si-Ni-San Attenuates Inflammation in the Liver Fibrosis Mouse Model

**FIGURE 5:**
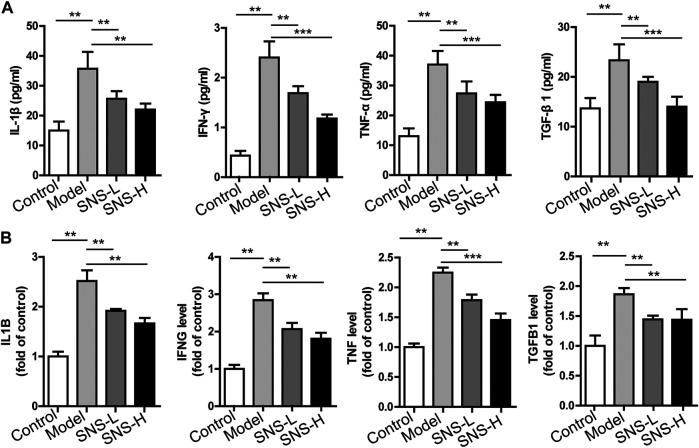
SNS attenuates inflammation in mice with liver fibrosis. Liver and serum samples were obtained from control and fibrotic mice administered water or SNS (5 g/kg and 10 g/kg) at the indicated times. **(A)** Secretion of IL-1β, IFN-γ, TNF-α and TGF-β1 in serum. **(B)** qRT-PCR analysis of f IL-1β, IFN-γ, TNF-α and TGF-β1 mRNA in liver using β-actin as the internal reference. Data are presented as mean ± SD (*n* = 6). *p < 0.05, **p < 0.01, ***p < 0.001 compared to control or model group.

Inflammation is closely associated with the development of liver fibrosis (Koyama and Brenner, 2017). Our network pharmacology results suggest that the beneficial effects of SNS on liver fibrosis are partially related to regulation of the inflammatory response. Accordingly, we explored the potential involvement of SNS in local and systemic inflammation. Compared with the control group, the serum contents of cytokines (such as TNF-α, IFN-γ, and IL-1β) together with their gene expression patterns in liver tissues of fibrotic mice were remarkably elevated. Interestingly, SNS application reversed the upregulation of these factors (Figures 5A,B). TGF-β1 can be excessively produced by various cell types and suggested to serve as the pro-fibrogenic factor in liver fibrosis (Beljaars et al., 2017). Consistently, our findings showed that the serum contents and mRNA levels of TGF-β1 in liver were increased in the model relative to the control group and SNS exposure led to suppression of TGF-β1 upregulation (Figures 5A,B). Our collective results suggest that SNS mitigates liver fibrosis through alleviating the proinflammatory milieu.

### Si-Ni-San Inhibits Pathological Microvessel and Angiogenesis-Associated Signaling Pathways in Liver Tissue of the Mouse Model

**FIGURE 6:**
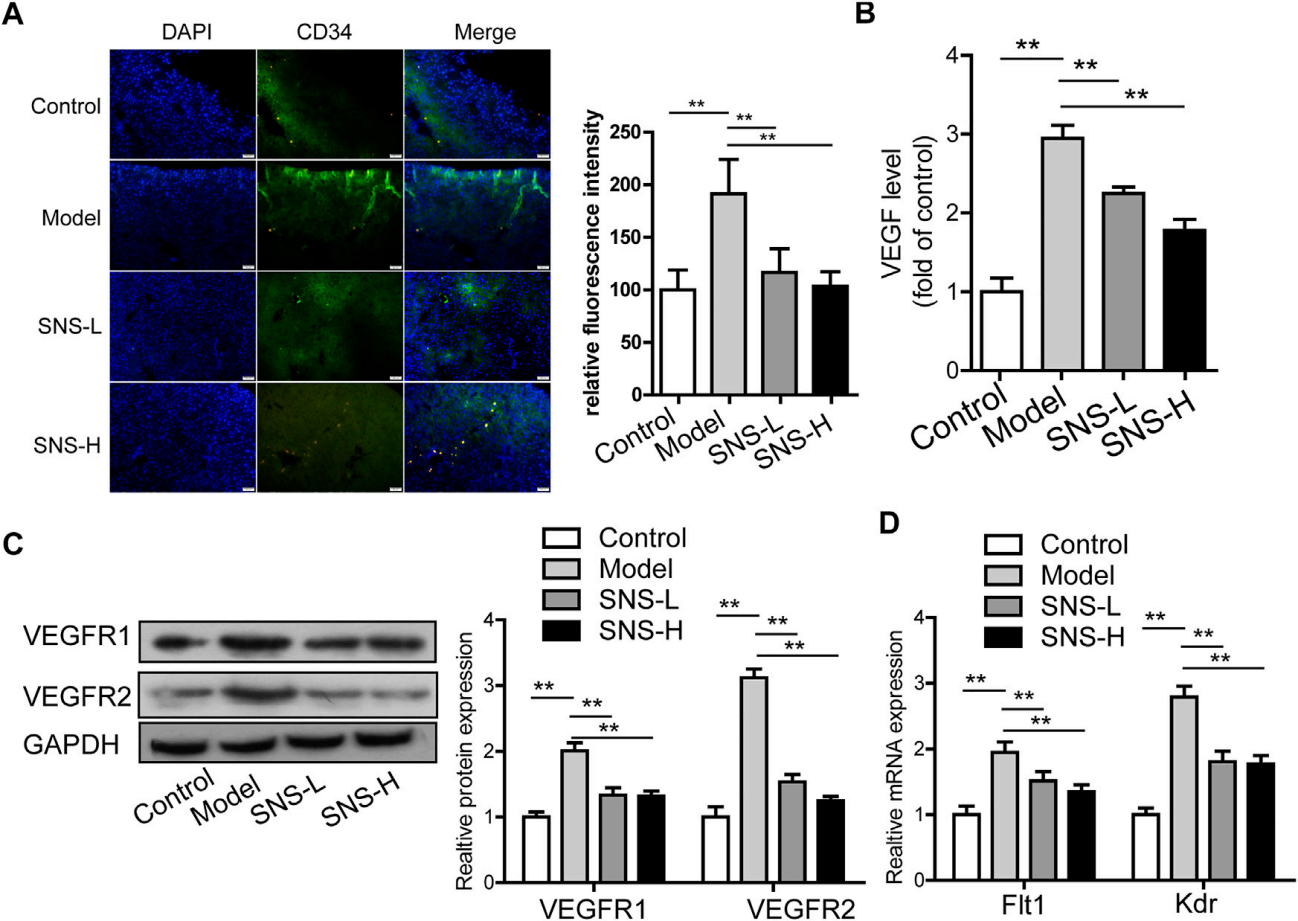
SNS inhibits pathological microvessel and angiogenesis-associated signaling pathways in liver tissue of mice with liver fibrosis. **(A)** Image analysis of fluorescence intensity of CD 34 (pathological microvessels) stained with IF in hepatic tissue of mice with CCl_4_-induced liver fibrosis. **(B)** Effect of SNS on VEGF expression in liver tissue. **(C)** Effects of SNS on VEGFR1 and VEGFR2 protein levels in liver tissue. **(D)** Effects of SNS on Flt1 and Kdr mRNA levels in liver tissue. Data are presented as mean ± SD (*n* = 6). *p < 0.05, **p < 0.01, ***p < 0.001 compared to control or model group.

To establish whether SNS affects pathological angiogenesis in liver tissues of the CCl_4_-induced liver fibrosis mouse model, CD34 levels were detected using the immunofluorescence assay. The number of fluorescence-stained cells and fluorescence intensity were remarkably increased after CCl_4_ stimulation and evidently reduced in the presence of SNS compared with the model group (*p* < 0.01; Figure 6A). Based on the above findings, we propose that SNS can partially suppress pathological hepatic angiogenesis in the CCl_4_-induced liver fibrosis mouse model. VEGF levels in liver tissues were subsequently determined in view of the modulatory effects of SNS on pathological microvessels. According to ELISA findings, CCl_4_ elevated the VEGF content in the model relative to the normal group while the VEGF content in SNS treatment groups was markedly reduced compared with the model group (*p* < 0.01) (Figure 6B). The results suggest SNS can effectively reduce the production of angiogenesis-related VEGF in mice with CCl_4_-induced liver fibrosis after long-term damage. We further determined the mRNA and proteins expression patterns of VEGF receptors (VEGFR1 and VEGFR2). Expression levels of both VEGFR1 and VEGFR2 were markedly decreased following SNS exposure (*p* < 0.01; Figures 6C,D).

In summary, SNS suppresses abnormal angiogenesis in tissues of mice with liver fibrosis, which is potentially associated with regulating expression of VEGF and its receptors.

### Si-Ni-San Protects Hepatic Parenchymal Cells From Apoptosis *in vivo* and *in vitro*

**FIGURE 7:**
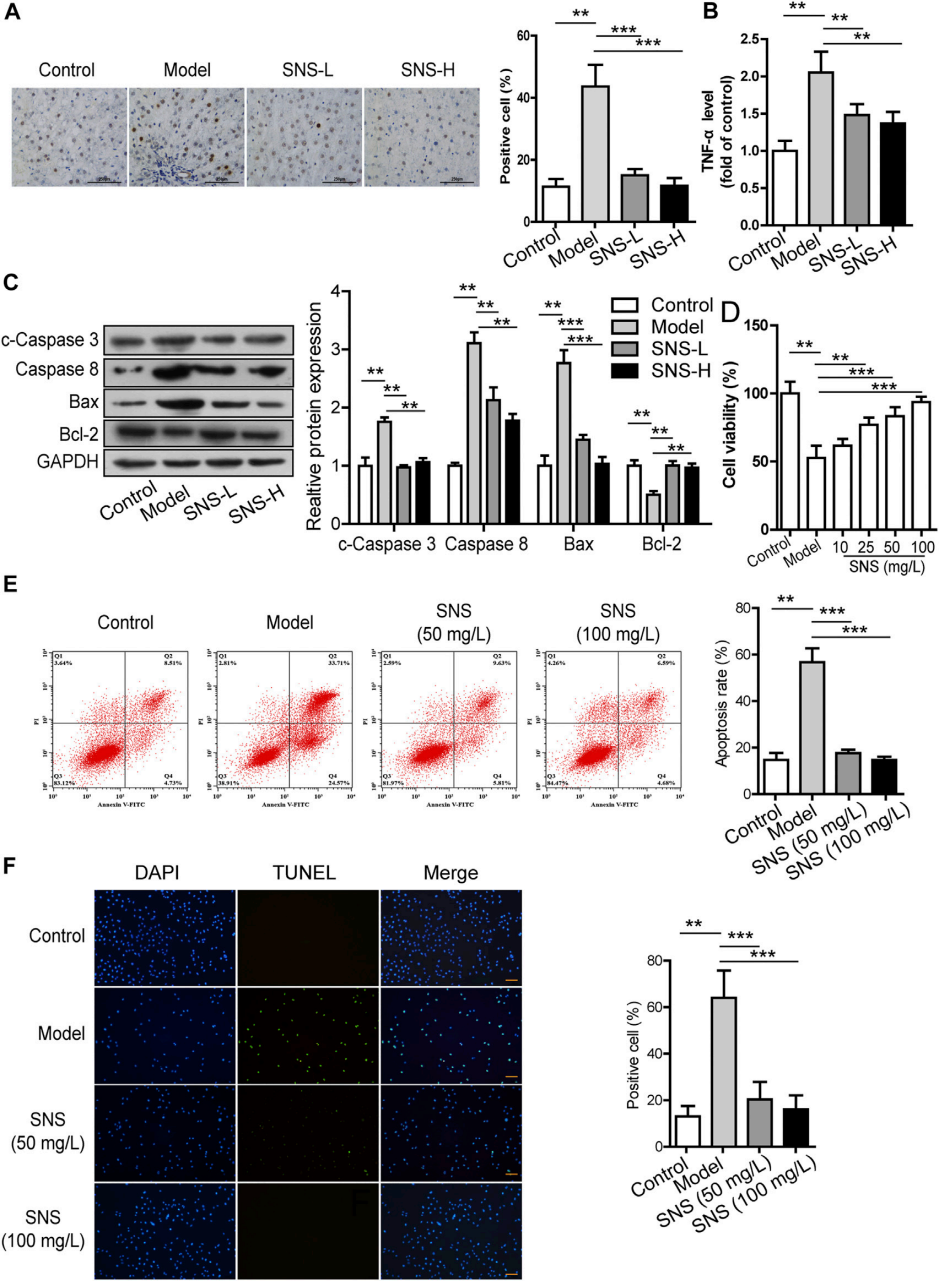
SNS protects hepatic parenchymal cells from apoptosis *in vivo* and *in vitro*. **(A)** TUNEL staining (100×) of liver tissues. **(B)** Effect of SNS on the TNF-α level in liver tissue. **(C)** Effects of SNS on cleaved caspase 3, caspase 8, Bax, and Bcl-2 in liver tissue. **(D)** Effects of SNS on viability of L02 cells stimulated with TNF-α and Act D. **(E)** Evaluation of apoptosis of L02 cells treated with SNS and TNF-α/Act D using the Muse Annexin V and Dead Cell Assay. **(F)** Evaluation of apoptosis of L02 cells treated with SNS and TNF-α/Act D *via* TUNEL staining. Data are presented as mean ± SD (*n* = 6). *p < 0.05, **p < 0.01, ***p＜0.001 compared to control or model group.

The TUNEL assay was conducted to examine the involvement of SNS in apoptosis of HPCs in the liver fibrosis mouse model. The proportion of TUNEL-positive HPCs was significantly increased in mice with CCl_4_-induced liver fibrosis, which was reversed in the presence of SNS (Figure 7A). SNS induced a marked decrease in TNF-α expression upregulated by CCl_4_ within liver tissues (*p* < 0.05; Figure 7B). Data from western blot analysis showed that SNS reduced the protein levels of cleaved caspase-3, caspase-8 (*p* < 0.05) and Bax (*p* < 0.01) but enhanced the Bcl-2 level (*p* < 0.05) in fibrotic liver tissues of mice, as shown in Figure 7C.

We further examined whether SNS plays a similar role in HPC apoptosis *in vitro*. To this end, the MTT assay was conducted to determine the viability of L02 cells after 24 h of SNS treatment. As shown in Figure 7D, L02 cell viability was remarkably decreased after TNF-α + Act D treatment (*p* < 0.001) and reversed following SNS exposure. The Muse Annexin V and Dead cell assay was conducted to evaluate apoptosis of L02 cells after 48 h of SNS treatment (Figure 7E). Relative to the control group, TNF-α + Act D treatment promoted L02 cell apoptosis (*p* < 0.01), which was suppressed by SNS. Similar results were obtained with the TUNEL assay (Figure 7F). In conclusion, SNS suppresses the apoptosis-inducing effects of stimulant on HPCs both *in vivo* and *in vitro*, possibly through regulation of apoptin expression and activation.

### Si-Ni-San Reverses Activation of Hepatic Stellate Cells Through Modulating PPAR-γ and NF-κB p65

**FIGURE 8:**
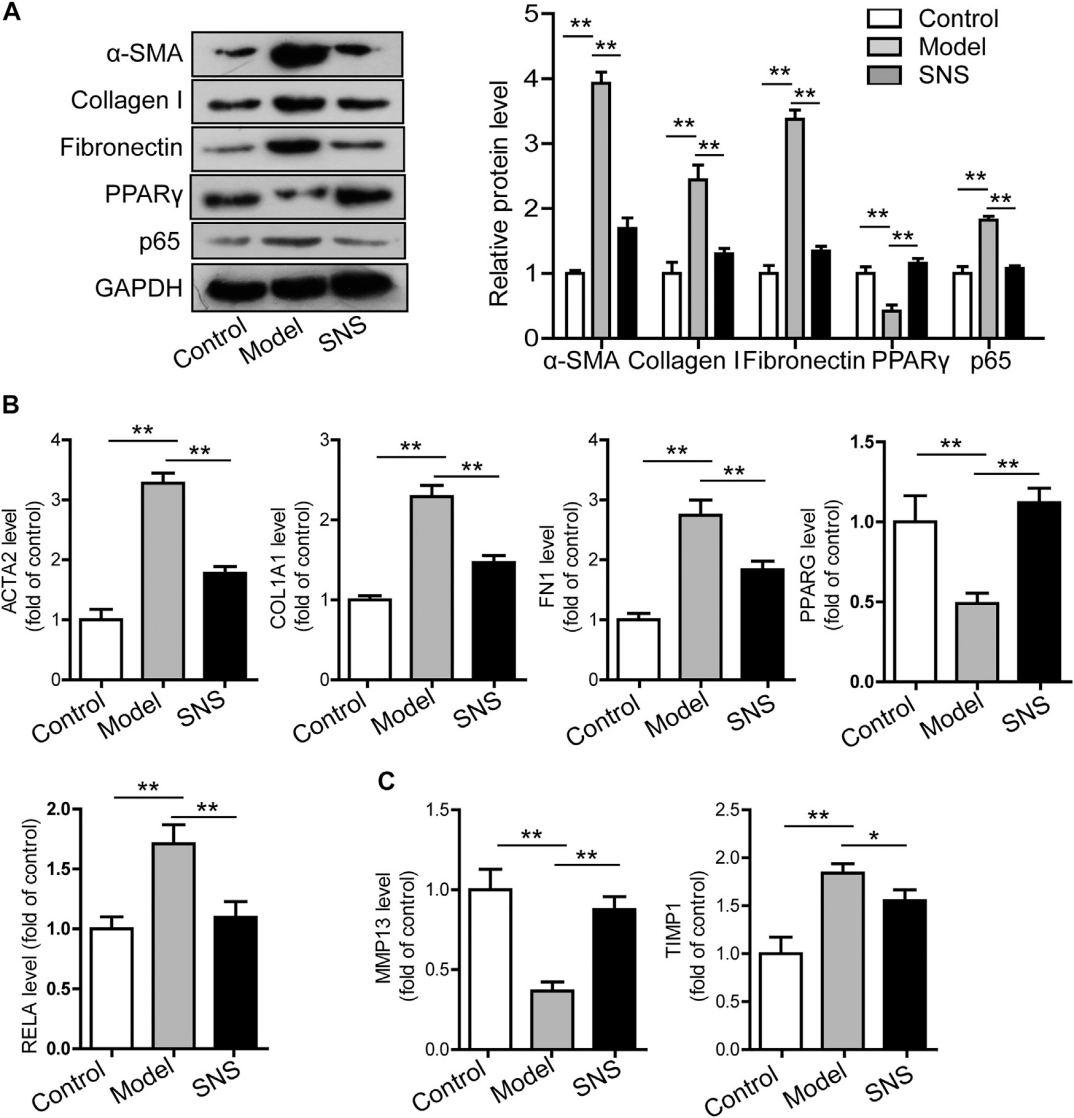
SNS reverses activation of HSCs through modulating PPAR-γ and NF-κB p65. **(A)** Effects of SNS on protein levels of α-SMA, collagen I, fibronectin, p65 and PPARγ in activated LX2 cells. **(B)** Effects of SNS on mRNA levels of ACTA2, COL1A1, FN1, PPARG and RELA in activated LX2 cells. **(C)** Effects of SNS on MMP13 and TIMP1 mRNA levels in activated LX2 cells.

Activated HSCs have been identified as critical hepatic fibrogenic effector cells, although other cells or processes also contribute significantly to this process. HSCs activated after liver injury proliferate and generate ECM. Our previous experiments demonstrated that SNS suppresses expression of the HSC activation marker α-SMA (Figure 1) and activation inducer TGF-β (Figure 5) in fibrotic liver tissues of mice in addition to collagen deposition (Figure 1). We further examined whether SNS directly acts in suppression of liver fibrosis with the aid of activated LX2 cells. Notably, SNS (50 mg/L) induced a decrease in collagen I, α-SMA and fibronectin mRNA and protein expression in activated LX2 cells (Figures 8A,B). Gene analysis data suggest that SNS reduces ECM generation through downregulating TIMP1 mRNA while upregulating MMP13 (Figure 8C). These findings indicate that SNS mitigates liver fibrosis by reversing HSC activation and altering the fibrogenesis-fibrolysis balance.

Upregulation of PPAR-γ in activated HSCs is suggested to suppress expression of collagen I, impede TGF-β1 signal transduction, inhibit cell proliferation and increase lipid droplets in the cytoplasm (Hamed et al., 2017). NF-κB p65 functions as a critical inflammation and fibrogenesis regulator in fibrotic liver disease (Wang Y.-H. et al., 2020). According to network pharmacological data, SNS positively affects liver fibrosis and HSC activation potentially through modulation of PPAR-γ and p65. Moreover, our results suggest that SNS promotes PPAR-γ mNRA and protein expression in activated LX2 cells concomitant with downregulation of NF-κB p65 (Figures 8A,B). PTGS2 and DPP4 were regulated by 96 and 80 of the 119 candidate compounds of SNS, respectively. No changes in these molecules in activated LX2 cells and liver tissue were observed (data not shown). Accordingly, we propose that SNS suppresses liver fibrosis and HSC activation, at least partly through regulating PPAR-γ and NF-κB p65 expression.

## Discussion

Liver fibrosis, a type of progressive disorder, is related to structural destruction of liver tissue, ECM deposition, and new pathological microvessel formation in liver as a result of pathological insult. Liver fibrosis is a pathological process frequently detected in most liver diseases, such as end-stage cirrhosis and hepatocellular carcinoma (HCC) (Sakurai and Kudo, 2013). Numerous therapeutic targets are currently under investigation with a view to identifying antifibrotic agents. Knowledge of these therapeutic targets may aid in: 1) decreasing the incidence of primary disease; 2) mitigating damage to HPCs through the use of hepatoprotectants; 3) suppressing the activation, contractility and fibrogenesis of myofibroblasts; 4) accelerating activated stellate cell apoptosis or reversion; and 5) inhibiting aberrant angiogenesis (Baglieri et al., 2019; Greuter and Shah, 2019; Manka et al., 2019; Chan et al., 2020). To date, the majority of treatments have only focused on a single cell, single cytokine or single signaling molecule without consideration of the overall complex process of liver fibrosis, leading to failure in achieving the expected clinical efficacy. Development of novel antifibrotic treatments involving two or more critical pathogenic targets and/or pathways is thus urgently required (Wang T. et al., 2020).

SNS is a common TCM prescription involving several components and targets. Data from the present study showed for the first time that SNS effectively reverses CCL_4_-induced liver fibrosis *in vivo* and facilitates recovery of liver function. We subsequently examined the candidate components and targets in SNS with the aid of network pharmacology analysis. As a result, 119 candidate compounds and 173 corresponding targets were found to be associated with SNS activity against liver fibrosis. Afterwards, the core active ingredients and targets of SNS for inhibiting liver fibrosis were screened through topological analysis. Several core components, such as Glycyrrhizic acid, Saikosaponin A, Paeoniflorin, and Naringin (Supplementary Table S8), which have been determined as the quality control substances in the Chinese Pharmacopoeia of the four single medicinal herbs constituting SNS and used for quality control of the experimental medicine in this study, have long been verified to significantly inhibit the diverse stimuli (CCl_4_ and dimethylnitrosamine)-induced liver fibrosis *via* multiple pathways. For example, Glycyrrhizic acid was verified to revere the occurrence of liver fibrosis through regulating the TGF-beta pathway and inhibiting parenchymal hepatic cell apoptosis (Liang et al., 2015; Zhou et al., 2016). Paeoniflorin can regulate the local immune-inflammatory responses of liver fibrosis pathology (Chen et al., 2012), while Naringin can suppress the NF-κB signal in hepatic stellate cells to regulate the TIMP-MMP13 pathway-mediated fibrogenesis/fibrolysis balance (Anuja et al., 2018). The core targets were further subjected to functional annotation and pathway enrichment analyses, which revealed that SNS predominantly modulates apoptosis, angiogenesis-related proliferation and migration of endothelial cells and immuno-inflammatory responses *via* TNF, VEGF, NF-κB, and MAPK pathways. Similarly, our experimental results showed that SNS mitigates inflammation in mice with liver fibrosis and protects against apoptosis of HPCs. Moreover, SNS directly suppressed aberrant angiogenesis, HSC activation and ECM accumulation, leading to inhibition of liver fibrosis.

Following induction of liver injury by CCl_4_ or other stimulators, a variety of cells generate proinflammatory cytokines, including TNF-α and IL-1β, to promote liver fibrosis (Weiskirchen and Tacke, 2016). In our study, SNS treatment reduced the serum and liver contents of TNF-α, IL-1β as well as IFN-γ in mice with liver fibrosis. The fibrotic pathology may additionally be related to increased expression of TGF-β1 that originally recruits fibroblasts and inflammatory cells at the injury site and subsequently promotes production of ECM and cytokines from these cells (Crosas-Molist et al., 2015). Overexpression of TGF-β1 in transgenic animals is reported to trigger spontaneous liver fibrosis (Kisseleva and Brenner, 2008; Wilson, 2015). Our results showed that SNS induced a decrease in serum and liver levels of TGF-β1 in mice with liver fibrosis, validating our target prediction that the inhibitory effects of SNS on fibrogenesis are related to TGF-β1 modulation. Based on the collective findings, we propose that the beneficial effects of SNS are potentially associated with alleviation of inflammation and ECM deposition with reduced TGF-β1 activity.

Angiogenesis and novel hepatic pathological microvessel formation in chronic liver disease facilitate progression of liver fibrosis (Li, 2020). Activation of HSCs and their conversion to myofibroblasts represent central links in liver fibrosis formation. In addition, activated HSCs produce proangiogenic factors to accelerate hepatic endothelial cell proliferation and migration, leading to promotion of fibrosis (Zadorozhna et al., 2020). In chronic liver disease, many newly formed pathological vessels are immature and useless. As a result, the vascular plexus is formed, which splits around the regenerating hepatocytes and impedes the exchange between hepatocytes and sinusoids. Therefore, regenerating hepatocytes cannot build a normal portal vein branch, thereby aggravating damage to hepatocytes (Kaur and Anita, 2013). In this regard, a vicious cycle is formed between liver fibrosis and pathological angiogenesis in the liver, whereby increased pathological angiogenesis triggers fibrotic progression (Elpek, 2015). VEGF and its receptors, VEGFR1 and VEGFR2, play important roles in this aberrant angiogenesis process (Yan et al., 2015). Our *in vivo* results suggest that SNS not only suppresses angiogenesis in liver tissues of CCl_4_-induced mice but also downregulates VEGF and its two receptors. The therapeutic effects of SNS on liver fibrosis may be associated with regulation of aberrant angiogenesis in liver under pathological conditions.

Apoptosis, also known as programmed cell death, is a critical biological process during liver fibrosis (Chakraborty et al., 2012). To date, the majority of studies on treatments for liver fibrosis have focused on inhibition of HPC apoptosis and promotion of HSC apoptosis (Marti-Rodrigo et al., 2020). Typically, HPC apoptosis not only results from liver injury but also serves as a vital inflammatory stimulus for HSC activation (Jaeschke, 2002). Apoptosis is also a self-controlled program that serves to maintain homeostasis. Numerous genes are involved in this process, including the Bcl-2 family, caspase family and TNF (Wei et al., 2018; Wang R. et al., 2019). The induction of fibrotic response may be attributable to persistent HPC apoptosis (Takehara et al., 2004). In addition, apoptotic hepatocyte DNA increases release of collagen, promoting HSC differentiation (Watanabe et al., 2007). Experiments from the current study showed that SNS significantly suppresses the proportion of apoptotic cells in liver tissues induced by CCl_4_ and regulates caspase activation and expression of multiple Bcl-2 family proteins. *In vitro*, SNS suppressed apoptosis of L02 cells induced by TNF-α combined with Act D.

MMP13 and TIMP1 are mainly produced by HSC cells and affected by cytokines such as IL-1β, TGF-β1 and TNF-α. Release of MMPs and TIMPs is stringently regulated depending on the HSC activation status (Hernandez-Gea and Friedman, 2011). Under conditions of chronic liver injury, MMP13 and TIMP1 levels are regulated in different ways, resulting in the formation of a positive feedback loop together with subsequent fibrogenesis. In activated HSCs, particularly those with increased TIMP-1 expression, MMP activity is suppressed and matrix proteins accumulate (Hemmann et al., 2007). As evident from our *in vitro* findings, SNS suppressed TIMP1 while upregulating MMP13, leading to recovery of the balance between MMP13 and TIMP1 in activated LX2 cells. SNS induced a significant reduction in the levels of the mesenchymal markers, α-SMA and collagen I, indicating that SNS alleviates liver fibrosis predominantly through mitigating ECM deposition.

NF-κB p65 is a transcription factor and critical inflammation and cell death regulator, which plays a key role in chronic liver disease (Ray, 2012). Activation of p65 in HSCs accelerates fibrogenesis through promoting the activity and survival of HSCs (Luedde and Schwabe, 2011). Interestingly, in our experiments, SNS downregulated NF-κB p65 mRNA and protein expression in activated LX2 cells, confirming data from functional annotation and pathway enrichment analyses supporting significant effects of SNS on the NF-κB p65 signal transduction pathway. In addition, quercetin and puerarin in SNS are reported to exert anti-inflammation and/or antifibrosis effects through suppressing the NF-κB pathway (Li et al., 2013; Li X. et al., 2016). SNS may therefore suppress fibrogenesis progression through regulating activation of NF-κB p65.

PPAR-γ, expressed in HSCs, plays an important role in maintaining cell quiescence (Hazra et al., 2004). PPAR-γ activation blocks TGF-β1/Smad signaling, suppresses HSC proliferation and causes apoptosis, both *in vivo* and *in vitro* (Han et al., 2020). As determined from our network analysis, several candidate components prevent liver injury, inflammation and HSC activation through enhancing PPAR-γ expression. Data from our validation experiments showed that SNS promotes PPAR-γ activity in activated LX2 cells. SNS-induced amelioration of inflammation, promotion of collagen accumulation and regulation of apoptosis may thus be partially achieved through regulation of PPAR-γ activation.

Although this study has explained the material basis and molecular mechanism of SNS against liver fibrosis to a certain extent, there are still some limitations to be futher solved. To begin with, some compounds of herbs in SNS were neglected in consideration of the inadequate data obtained from existing databases and laboratory findings. Secondly, the influence of absolute content of each compound in SNS or the serum and fibrotic liver tissue distribution concentrations on its effect was ignored. Thirdly, this study only illustrated the regulatory effect of SNS on targets, but it did not explain the regulatory effects and patterns of combined actions (synergy, antagonism or additive) of the complex mixture of key active ingredients. As a result, in future research, we aim to extensively examine: 1) the enrichment degrees and contents of screened core active ingredients in the blood or liver tissues of experimental animals or patients through UPLC-MS to further confirm the core active ingredients in SNS against liver fibrosis; 2) the regulatory effect and patterns of combined actions of core active ingredients on the screened core targets and signaling pathways in patients, animal models and *in vitro* experiments using molecular biological technology.

## Data Availability

The original contributions presented in the study are included in the article/Supplementary Material, further inquiries can be directed to the corresponding authors.
