# Supplementary figures and images for: Network Pharmacological Analysis and Experimental Validation of the Mechanisms of Action of Si-Ni-San Against Liver Fibrosis
Source: Front Pharmacol. 2021 Jul 1;12:656115. doi: 10.3389/fphar.2021.656115 (PMC8281251; doi:10.3389/fphar.2021.656115)

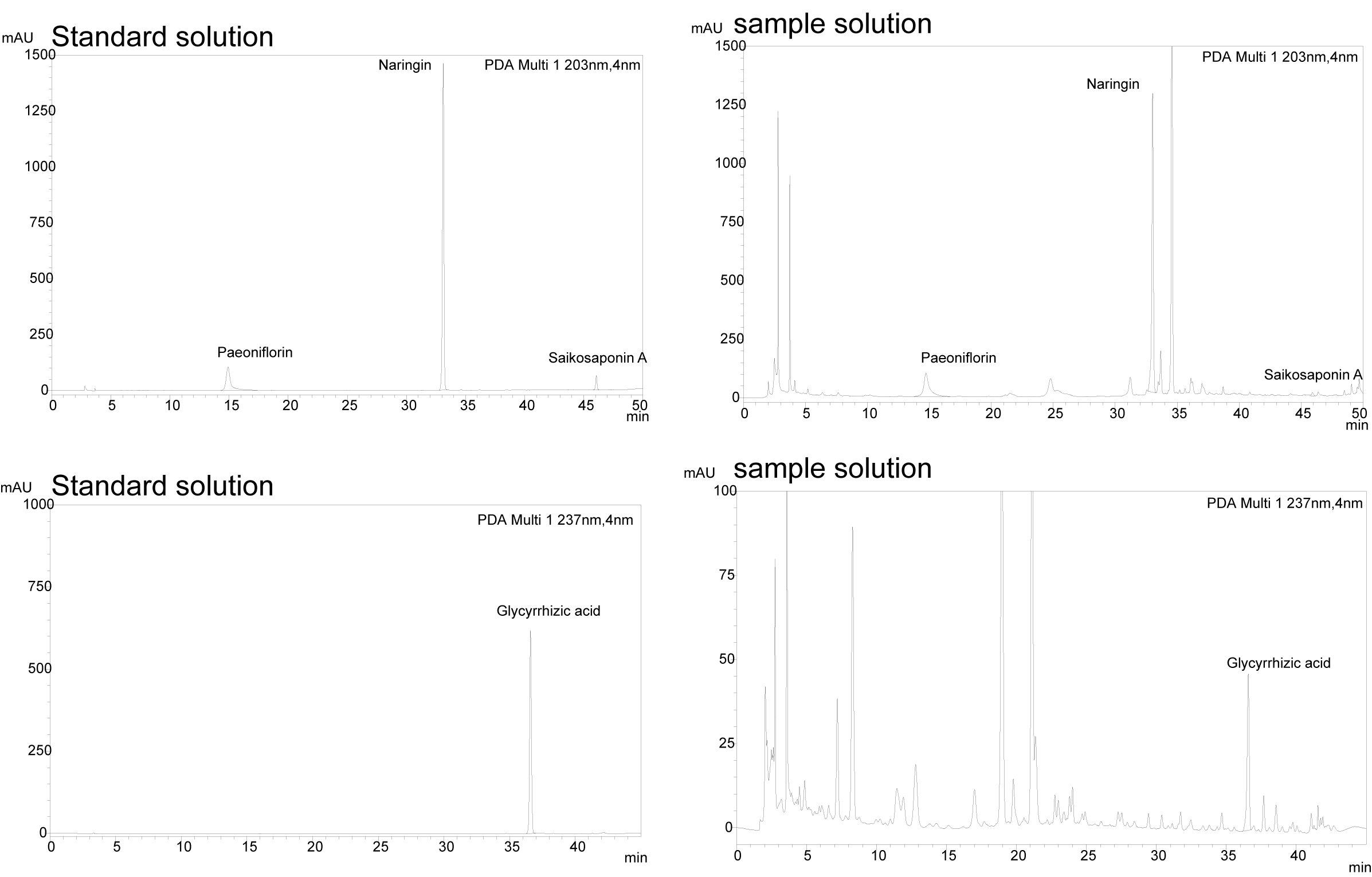

Supplement: Supplementary file 4 [file Image1.tif]
