# Supplementary material for: Network Pharmacological Analysis and Experimental Validation of the Mechanisms of Action of Si-Ni-San Against Liver Fibrosis
Source: Front Pharmacol. 2021 Jul 1;12:656115. doi: 10.3389/fphar.2021.656115 (PMC8281251; doi:10.3389/fphar.2021.656115)
Supplement: Supplementary file 11 [file DataSheet1.DOCX]

**Supporting Information**

**Network Pharmacology and Experimental Validation of the Mechanisms of Action of Si-Ni-San Against Liver Fibrosis**

Siliang Wang^1#^, Cheng Tang^2#^, Heng Zhao^3#^, Peiliang Shen^4^, Chao Lin^5^, Yun Zhu^1^, Dan Han^1*^

^1^ Department of Pharmacy, Drum Tower Hospital Affiliated to the Medical School of Nanjing University, Nanjing, China.

^2^ Department of Respiratory Medicine, Affiliated Hospital of Integrated Traditional Chinese and Western Medicine, Nanjing University of Chinese Medicine, Nanjing, China.

^3^ Department of Endocrinology, Jinling Hospital, Jinling Hospital, Medical School of Nanjing University, Nanjing, China.

^4^ School of Pharmacy, School of Medicine & Holistic Integrative Medicine, Nanjing University of Chinese Medicine, Nanjing, China.

^5^ School of Medicine & Holistic Integrative Medicine, Nanjing University of Chinese Medicine, Nanjing, China.

^#^ Equal contribution

^*^ Correspondence and requests for materials should be addressed to Siliang Wang (Email: wsl_dth@126.com) and Dan Han (Email: handanjelly@126.com)

Fig S1. HPLC chromatographic diagrams for determinations of Glycyrrhizic acid, Saikosaponin A, Paeoniflorin and Naringin in SNS freeze-dried powder.

Table S1. Hepatic fibrosis grading system

Table S2. Gene primers applied in qRT-PCR analysis

Table S3. Potential pharmacodynamic components of SNS

Table S4. Potential pharmacodynamic targets of SNS

Table S5. Known liver fibrosis-related targets

Table S6. Pharmacodynamic components of SNS sharing 173 potential targets with known liver fibrosis-related targets

Table S7. Degree values of nodes in the candidate component-target network of SNS associated with liver fibrosis

Table S8. Core pharmacodynamic components of SNS with activity against liver fibrosis

Table S9. Core targets of SNS in the treatment of liver fibrosis
